# AgriGAN: unpaired image dehazing via a cycle-consistent generative adversarial network for the agricultural plant phenotype

**DOI:** 10.1038/s41598-024-65540-0

**Published:** 2024-07-01

**Authors:** Jin-Ting Ding, Yong-Yu Peng, Min Huang, Sheng-Jun Zhou

**Affiliations:** 1 School of Information and Electrical Engineering, Hangzhou City University, Hangzhou, 310015 Zhejiang China; 2 Zhejiang Academy of Agricultural Sciences, Hangzhou, 310021 Zhejiang China

**Keywords:** Agricultural images, Generative adversarial networks, Image dehazing, Information extraction, Electrical and electronic engineering, Applied mathematics

## Abstract

Artificially extracted agricultural phenotype information exhibits high subjectivity and low accuracy, while the utilization of image extraction information is susceptible to interference from haze. Furthermore, the effectiveness of the agricultural image dehazing method used for extracting such information is limited due to unclear texture details and color representation in the images. To address these limitations, we propose AgriGAN (unpaired image dehazing via a cycle-consistent generative adversarial network) for enhancing the dehazing performance in agricultural plant phenotyping. The algorithm incorporates an atmospheric scattering model to improve the discriminator model and employs a whole-detail consistent discrimination approach to enhance discriminator efficiency, thereby accelerating convergence towards Nash equilibrium state within the adversarial network. Finally, by training with network adversarial loss + cycle consistent loss, clear images are obtained after dehazing process. Experimental evaluations and comparative analysis were conducted to assess this algorithm's performance, demonstrating improved accuracy in dehazing agricultural images while preserving detailed texture information and mitigating color deviation issues.

## Introduction

High-throughput, automated, high-resolution plant phenotypic information collection and analysis technologies are critical for accelerating plant improvement, breeding, yield enhancement, and pest control^1,2^. Plant phenotypic information collection and analysis techniques are used to parse genomic information. The quantitative study of complex traits related to growth, yield, and adaptation to biotic or abiotic stress is an important way to build plant growth models and collect phenotypic datasets of cucumbers, which can help fill the gap between genomic information and plant phenotypic plasticity. To acquire phenotypic information, researchers measure the specific plant height, canopy temperature, and other parameters of each breeding plot manually^3,4^. However, the manual measurement method has disadvantages, such as strong randomness, low efficiency, high time consumption, and a small amount of information obtained. With the development of artificial intelligence, machine vision in agriculture can be used to quickly obtain phenotypic information about cucumbers^5,6^. Furthermore, based on phenotypic information, cucumbers s can be effectively monitored dynamically, which is an important means of implementing precision agriculture. However, in practical applications, the images collected in foggy weather are blurry, making it difficult to accurately extract image features. Therefore, a dehazing algorithm for images in the agricultural field is important.

In the realm of computer vision^7–9^, the existing haze removal methods can be broadly classified into the following three categories: image enhancement algorithms, model-based haze removal algorithms^10,11^, and deep learning approaches^12–14^. The traditional model-based algorithm analyzes the causes of haze and restores the original haze-free image by establishing an atmospheric scattering model to understand the physical principle behind image degradation. While these algorithms are less prone to significant loss of image features, their efficacy in practical applications is often limited. For instance, He^15^ introduced a dark channel prior (DCP) algorithm, leveraging the observation that outdoor images tend to contain numerous dark pixels, yielding promising results. However, this method may not be suitable for scenes where a substantial part of the image is covered by light or similar objects. To address this limitation, Jin^16^ proposed an image dehazing algorithm based on guided filtering and adaptive tolerance, specifically targeting distortion in the sky area. Despite these advancements, traditional algorithms still face limitations in their effectiveness, rendering them unsuitable for all scene images.

Deep learning has made major strides in various fields, and its use to solve the problem of haze-free has achieved decent results. Li^17^ proposed the AOD-Net, which directly generates clear images through a lightly convolutional neural network combined with an atmospheric scattering model. Xiao^18^ proposed an image conversion algorithm for hazy scenes based on generative adversarial networks, which realizes the mutual conversion between the hazy image and the haze-free image. Zhu^19^ proposed a cycle-consistent adversarial network (CycleGAN) that doesn’t require paired images, and it provides solutions for the problem of scarce datasets. Zhao^20^ proposed a double-discriminator cycle-consistent adversarial network (DD-CycleGAN) for dehazing road scenes. Only a small amount of unpaired data is needed, greatly reducing the difficulty of data collection. As we know, deep learning uses convolution, pooling, and other methods to extract key features in images to solve various problems, but it does not add various models calculated by traditional algorithms. This causes two problems: (1) The network learns the features obtained using traditional algorithms but consumes much time and computing resources. (2) The network does not learn the features obtained using the traditional algorithm, which reduces the accuracy of a certain aspect of the reconstructed image. Given the defects of the dehazing model of the deep learning algorithm, it is necessary to consider the combination of deep learning and traditional methods.

Although the above studies have achieved good results, agricultural images have distinct characteristics, such as images overlapping between cucumbers leaves and leaves, leaves and fruits overlapping, and overlapping with shelves. Furthermore, because of the pattern of plant growth, it is theoretically impossible to obtain hazy and haze-free images in the agricultural field at the same time. Therefore, it is not ideal to apply the above algorithm directly to the dehazing effect of farmland images. However, researchers are still trying to perform dehazing research in the field of agriculture.

Wei^21^ proposed a new image dehazing method based on DCP theory and the interval interpolation wavelet transform for the degradation of image quality collected in hazy weather conditions. It combines the DCP model and the interval interpolation wavelet transform to restore the color features of the scene and make the image clearer. Zhang^22^ first proposed an improved image dehazing approach based on the DCP method and determined optimal enhancement parameters. The clarity of remote-sensing images is often affected by clouds and chaotic media in the atmosphere. Image dehazing can be achieved through the DCP, but there is always a brightness distortion problem after image dehazing. Still, DCP can be used for image dehazing in remote sensing. Fan^23^ proposed a dehazing method for agricultural images based on a super-pixel-level DCP and an adaptive tolerance mechanism to improve the guided filtering algorithm, aiming at the slow operation speed and poor applicability of the traditional DCP algorithm.

For precision agriculture, the purpose of dehazing is not only to improve the clarity of images but also to help computers obtain more reliable phenotypic information and to promote agricultural breeding analysis, growth management, and harvest acquisition. Therefore, our new algorithmic framework combines the atmospheric scattering model with CycleGAN, which adopts an asymmetric dual-discriminator structure to obtain high-quality agricultural scene images with unpaired data.

## Related works

### Dehazing of agricultural images

Agricultural images contain colorful targets with sharp edges and texture details. For precision agriculture, the purpose of dehazing is not only to improve the clarity of the image but also to restore the true color and avoid information bias. In 2019, Gao^24^ proposed a farmland image dehazing algorithm based on the Shannon-Cosine wavelet combined with the fine integration method, and the restored image showed excellent clarity. In 2021, Fan^23^ proposed a dehazing method for agricultural images based on the super-pixel-level dark channel before improving the guided filtering algorithm on agricultural robot visual images. The method obtained high-quality agricultural scene images, and the color information and clear details of the original image were preserved. The algorithm also retained the color information of the original image and provided a research basis for the analysis of cucumbers phenotype information in precision agriculture.

### GAN

A GAN^25^ consists of two networks: the generator (G) and the discriminator (D). The generator generates fake images to fool the discriminator, and the discriminator learns to distinguish between real and fake images. The goal of G is to maximize the error rate of D, and the goal of D is to maximize the accuracy of discrimination. The training process is an adversarial game that pits G and D against each other. The process can be expressed as follows:

1 $$ \mathop {\min }\limits_{G} \mathop {\max }\limits_{D} V(D,G) = E_{{x\sim p_{data} }} [\log D(x)] + E_{{z\sim p_{z} (z)}} [\log (1 - D(G(z)))] $$

Unlike the parameter updates of traditional deep learning, that of a GAN uses back-propagation of the discriminator, and the parameter updates of traditional deep learning only come from sample data. In this scenario, image-to-image translation is a typical task in which a GAN plays an important role. GAN-based image translation refers to converting from one set of domain images to another without changing the subject of the image, while the essence of dehazing is to convert one set of hazy images to another, so dehazing can be used in image translation methods. In unpaired image translation, CycleGAN^19^, DualGAN^26^, and DiscoGAN^27^ all use unsupervised training, which solves the problem of the paired data required by the supervised network. Moreover, CycleGAN, DualGAN, and DiscoGAN all use cycle consistency loss to save the key information of the input and translated images and realize the translation of images in the case of unpaired data. However, the three considerably differ in implementation. CycleGAN adopts the style transfer architecture of Johnson and adds the residual network to the network to stabilize network training, which improves the quality of generated images. In contrast, DualGAN only uses the autoencoder structure, but it achieves a significant translation effect. DiscoGAN draws on the generator of Pix2pix GAN^28^, which uses the U-net^29^ symmetric structure to join the residual network. It can perform a variety of structural comparisons to prove the effectiveness of the network. However, the above algorithms have been improved for the characteristics of dehazing, so we propose AgriGAN for agricultural dehazing based on CycleGAN.

## Proposed method

### Dual-encoding generator based on U-net structure

It has been proved in the relevant knowledge of physics and optics that the algorithm of image dehazing based on the atmospheric scattering model is effective. The atmospheric scattering model uses the propagation properties of light in the atmosphere to build a physical model. The model formula is as follows:

2 $$ I\left( x \right) = J\left( x \right)t\left( x \right) + A\left[ {1 - t\left( x \right)} \right] $$

where J(x) is the image without haze, I(x) is the image with haze, A is the atmospheric light value, and t(x) is the transmittance. Now, the known condition is that for the image with fog I(x), we take the value with the highest brightness in the original image as the A value. If J(x) is solved, multiple sets of solutions will be available without any restriction.

The current method of generating an adversarial network to solve the problem is as follows: Take I(x) as the input of the generator, take J(x) as the output of the generator, and make the generator obtain a haze-free image through the adversarial training of the generator and the discriminator. Given the aforementioned problems with this approach, Tu et al. proposed the generative adversarial network dehazing algorithm combined with the atmospheric scattering model to achieve high-precision dehazing of paired images. However, in real life, paired image data collection is characterized by high time consumption and cost. Therefore, the proposed non-matching image dehazing algorithm is necessary.

**Figure 1:**
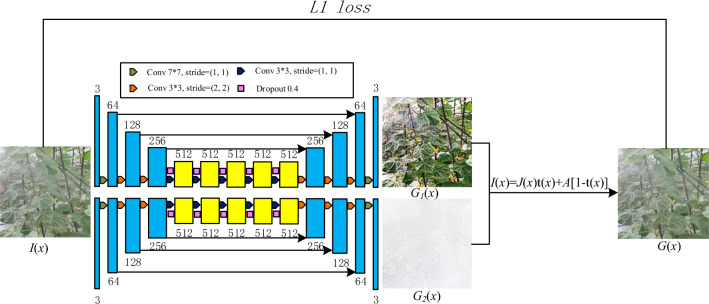
The structure of the generator.

The generator of the model, G2, is shown in Fig. 1, in which the encoder downsamples the hazy image, I(x), and after the downsampling, the decoder is used to decode the encoded data. Encoder 1 is similar to the existing deep learning method, which aims to obtain the haze-free image, J(x), and encoder 2 is used to fit the transmittance, t(x). According to the atmospheric scattering model, I(x) and t(x) have a relationship, so it is feasible to use I(x) to generate t(x). The real haze-free image and the transmittance are denoted by J(x) and t(x) in the algorithm, respectively. The generated haze-free image and transmittance are denoted by G1(x) and G2(x), respectively. G1(x) and G2(x) are restored to the hazy image, G(x), through the atmospheric scattering model. Take the L1 distance between the G(x) and the real foggy image, J(x), as the difference. Finally, the network transmits the difference back to the network to improve the efficiency of the network to generate images. In the structure of the generator, we refer to the U-net^29^ network structure proposed by Ronneberger, which copies the features of the same dimension in the downsampling to the upsampling, which reduces the probability of generating poor images. The downsampling of the network uses the 3 × 3 convolutional layer and the maximum pooling layer to collect features from the image. In the upsampling, deconvolution is used to restore the image scale. The structure of the generator network is shown in Fig. 1, while the parameters of the generator network are shown in Table 1.

**Table 1:** The parameters of the generator network.

^Kernel size^	^Kernel size^	^Stride^	^Output^
^2^*^Conv Block^	^7^	^1^	^64^
^2^*^Conv Block^	^3^	^2^	^128^
^2^*^Conv Block^	^3^	^2^	^256^
^2^*^Residual Block^	^3^	^1^	^512^
^2^*^Residual Block^	^3^	^1^	^512^
^2^*^Residual Block^	^3^	^1^	^512^
^2^*^Residual Block^	^3^	^1^	^512^
^2^*^Residual Block^	^3^	^1^	^512^
^2^*^Deconv Block^	^3^	^2^	^256^
^2^*^Deconv Block^	^3^	^2^	^128^
^2^*^Deconv Block^	^3^	^2^	^64^
^2^*^Deconv Block^	^7^	^1^	^3^

### Discriminator based on detail-holistic consensus discrimination

Unlike traditional generative adversarial networks, we designed the dual-discriminator structure to find features at different levels in images. In the algorithm, the two generators are called the detailed discriminator and the structure discriminator. The structure discriminator captures the overall outline of the image and other information to help the generator enhance the fitting of the overall structure of the image, while the detailed discriminator focuses on capturing the texture, pixels, and other detailed information to enhance the fitting of the details of the generated image by the generator, making the generated image more realistic.

**Figure 2:**
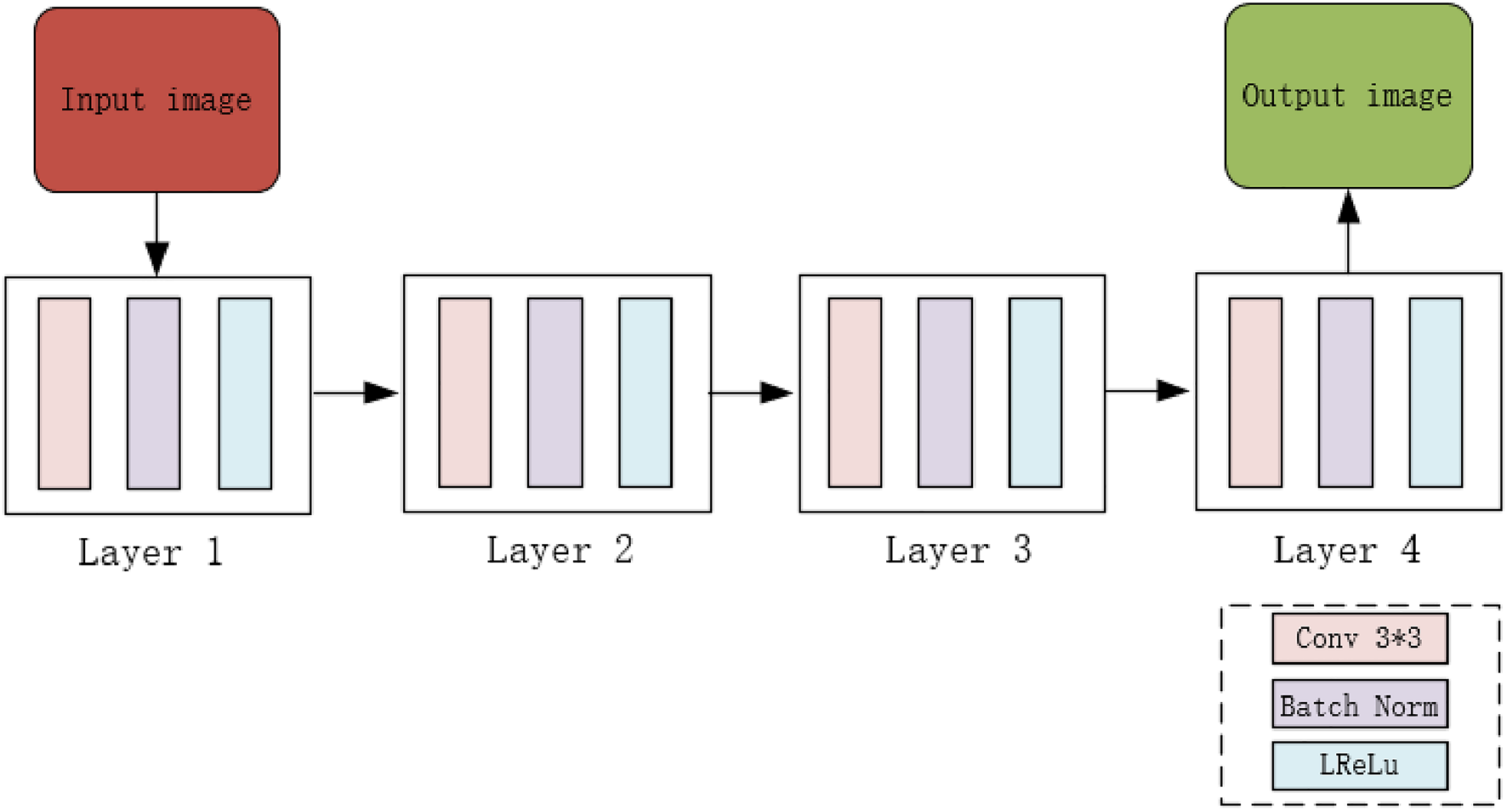
Detailed discriminator structure.

The detailed discriminator has a four-layer network structure. The four-layer network is trained by the Conv-Batch Normalization-Leaky ReLU layer repeatedly. Finally, the learned features are input into the sigmoid function through a 1 × 1 convolution to calculate the results. The overall structure of the detailed discriminator is shown in Fig. 2.

**Figure 3:**
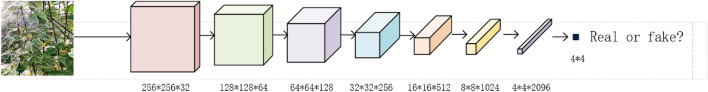
Overall discriminator structure.

The structure discriminator uses a traditional convolutional neural network, which takes the entire image as input and outputs a 4 × 4 matrix block after seven layers of convolution-pooling and a sigmoid function. When training the network's ability to discriminate real images, input the real image and label 1 into the discriminator network, and when training the network's ability to discriminate on converted images, input the converted image and label 0 into the discriminator network. During the test, the unlabeled image is input into the network, and the network outputs the value of 0–1 to form the discriminant information. The structure discriminator in the network structure is shown in Fig. 3.

The dual-discriminator structure is used to solve the mode collapse problem of the traditional GAN and provide stable feedback for the generator. Moreover, through the concept of detail and structure, the structure guides the generator to output the authenticity of the image and enhance the generation server network performance.

### Loss function

Adding network loss is a key means of training the network. For the translation-limited problem, choosing an appropriate loss function is the key to solving the problem. We adopt the concept of adversarial loss + cycle-consistent loss to improve the loss function for the proposed network.

The adversarial loss of the network is given as follows:

3 $$ L_{GAN} \left( {G_{1,2} ,D_{Y} ,X,Y} \right) = E_{{y\sim p_{data} }} \left( y \right)\left[ {\log D_{{Y_{1} Y_{2} }} \left( y \right)} \right] + E_{{x\sim P_{data} }} \left( x \right)\left[ {\log \left( {1 - D_{{Y_{1} Y_{2} }} \left( {G_{Y} \left( x \right)} \right)} \right)} \right] $$

4 $$ L_{GAN} \left( {F_{1,2} ,D_{X} ,X,Y} \right) = E_{{x\sim p_{data} }} \left( x \right)\left[ {\log D_{X} \left( x \right)} \right] + E_{{y\sim P_{data} }} \left( y \right)\left[ {\log \left( {1 - D_{X} \left( {F_{X} \left( y \right)} \right)} \right)} \right] $$

Because of the unpaired dataset, using the traditional GAN loss function does not make the network generates images of the domain Y corresponding to the domain X, so the cycle-consistent loss is added to the network. The cycle-consistent loss formula is as follows:

5 $$ L_{cycle} \left( {G_{1} ,F_{1} } \right) = E_{{x\sim p_{data(x)} }} \left[ {\left\| {F_{1} \left( {G_{1} \left( x \right)} \right) - x} \right\|} \right] $$

6 $$ L_{cycle} \left( {G_{2} ,F_{2} } \right) = E_{{x\sim p_{data(x)} }} \left[ {\left\| {F_{2} \left( {G_{2} \left( x \right)} \right) - x} \right\|} \right] $$

where G_1_ and G_3_ are conversions from field X to field Y, and G_2_ and G_4_ are conversions from field Y to field X. Therefore, the complete objective function is as follows:

7 $$ L\left( {G_{1,2} ,G_{3,4} ,D_{X} ,D_{Y} } \right) = L_{GAN} \left( {G_{1,3} ,D_{Y} ,X,Y} \right) + L_{GAN} \left( {G_{2,4} ,D_{X} ,X,Y} \right){ + }\lambda \left( {L_{cycle} \left( {G_{1} ,F_{1} } \right) + L_{cycle} \left( {G_{2} ,F_{2} } \right)} \right) $$

where $\lambda$ is the hyperparameter and the weight of the cycle-consistent loss.

### Algorithm framework

**Figure 4:**
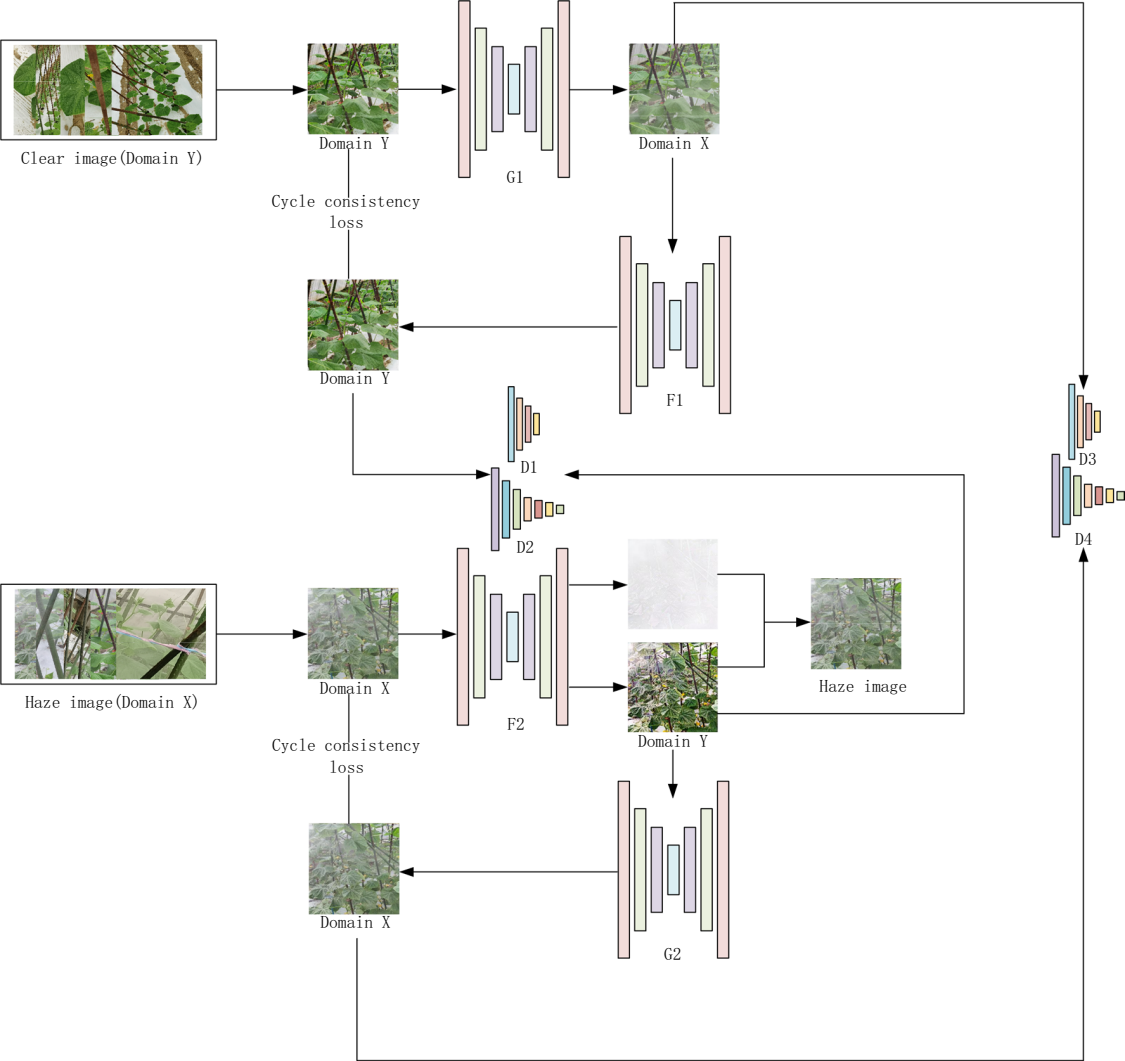
The structure of the AgriGAN.

To improve the dehazing performance of images effectively, this paper improves the generator, discriminator, and loss function of the original network by the atmospheric scattering model, dual decoder structure, and dual discriminator structure. The algorithm flow is shown in Fig. 4, where the haze-free image, y/hazy image, x, is converted into the hazy image, G_1_(y)/haze-free image, G_2_(x), through generator G_1_ and generator G_2_.

The hazy image, G_1_(y)/haze-free image, G_2_(x), are restored to the haze-free image, F1(G1(y))/hazy image, F2(G2(x)), by generators F1 and F2. Here, F1(G1)y))≈y, and F2(G2(x))≈x. The generator G2 generates the haze-free image and a transmittance map, t (x), at the same time. The hazy image is restored by the atmospheric scattering model $I\left( x \right) = J\left( x \right)t\left( x \right) + A\left[ {1 - t\left( x \right)} \right]$. Here, I (x) is a hazy image, J (x) is a haze-free image, t (x) is a transmittance map, and A is the atmospheric light value, the latter of which is replaced by the maximum light value in the image. Discriminators D1, D2, D3, and D4 are used to distinguish whether an image contains fog. D1 and D2 are used to determine whether a haze-free image is a real image or a generated image, while D3 and D4 are used to determine whether the haze-free image is a real image or a generated image. The structure of the algorithm is shown in Fig. 4.

The hazy image undergoes processing by generator F2 to produce the haze-free image and transmittance. Subsequently, the haze loss is computed by comparing the atmospheric scattering model with the hospital's hazy image. The haze-free image is then transformed back into the hazy image via generator G2 to establish consistency in loss. Generator F1 corresponds to G1. Discriminators D1 to D4 ascertain the presence of haze in the image.

## Experimental results and analysis

**Figure 5:**
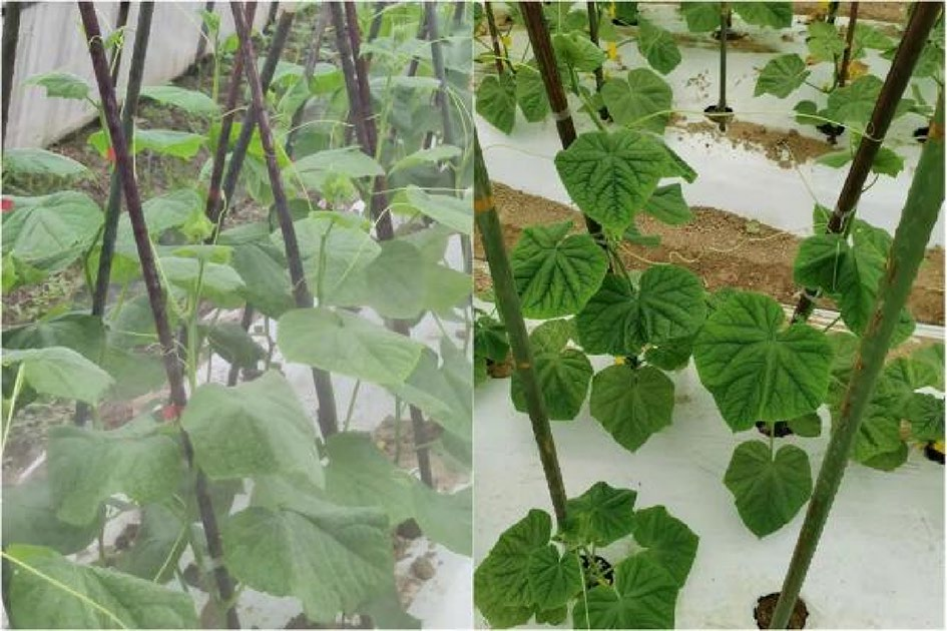
The dataset of our experiment.

To verify the advantages of the proposed method in the image dehaze algorithm, the research team selected a mountain village in Hangzhou, Zhejiang, as the experimental research object. Due to local climate conditions and other reasons, a series of noises such as water haze will appear when the water vapor is relatively sufficient. Therefore, the research team selected the images collected when water haze appeared to create the dataset. The training dataset contains 407 images of fogless plants and 479 images of haze plants. The test dataset contains 51 images of haze-free plants and 49 images of haze plants. There is no mutual match between hazy images and haze-free images, as shown in Fig. 5.

The experimental environment was the Tiankuo W580-G20 server, the CPU processor was E5-2600V3, the GPU processor was 2080ti, the experimental tool was Python 3.6.5, the algorithm was implemented with TensorFlow framework, and Slim library and Opencv library were installed to simplify code redundancy.

For the consistency of fixed data, each image was resized to 256 × 256 × 3, and all networks were optimized by the Adam algorithm. The learning rate of the generator and discriminator for the first 5,000 generations was 1e-4, and that of the last 5000 generations was reduced to 0. The loss function and training time were recorded once every 10 iterations of 10,000 iterations.

### Algorithm evaluation index

For the image definition index, the energy gradient function (EGF) was used to evaluate the image definition:

8 $$ D\left( f \right) = \frac{{\sum\nolimits_{y} {\sum\nolimits_{x} {\left( {\left| {f\left( {x + 1,y} \right) - f(x,y)} \right|^{2} + \left| {f(x,y + 1) - f\left( {x,y} \right)} \right|^{2} } \right)} } }}{256 \times 256} $$

where f (x, y) is a point in the image, f (x, y + 1) is a point below f (x, y), and f (x, y + 1) is a point on the right side of the image.

(2) Fréchet inception distance (FID).

FID extracts the feature extraction layer of the inception network as the feature extraction function. The function calculates the distribution mean and variance between the real distribution, P_data_, and the generated distribution, P_z_, and it calculates the distance between the real distribution and the generated distribution by obtaining the mean and variance information:

9 $$ FID\left( {x,z} \right) = \left\| {\mu_{x} - \mu_{z} } \right\|_{2}^{2} + Tr\left( {\mathop \Sigma \limits_{x} + \mathop \Sigma \limits_{z} - 2\left( {\mathop \Sigma \limits_{x} \mathop \Sigma \limits_{z} } \right)^{\frac{1}{2}} } \right) $$

where μ_x_ and μ_z_ represent the mean–variance value of the real distribution and the generated distribution, respectively.

In sum, the smaller the FID value, the closer the generated distribution is to the real distribution, and the closer the generated image is to the real image.

### Comparison with the state-of-the-art algorithms

To verify the effectiveness of the proposed algorithm, we compared it with the MSCNN-HE algorithm proposed by Cao, the DDcycleGAN proposed by Zhao, the DualGAN proposed by Lu, and the DeHazeNet^30^ algorithm proposed by Cai.

**Figure 6:**
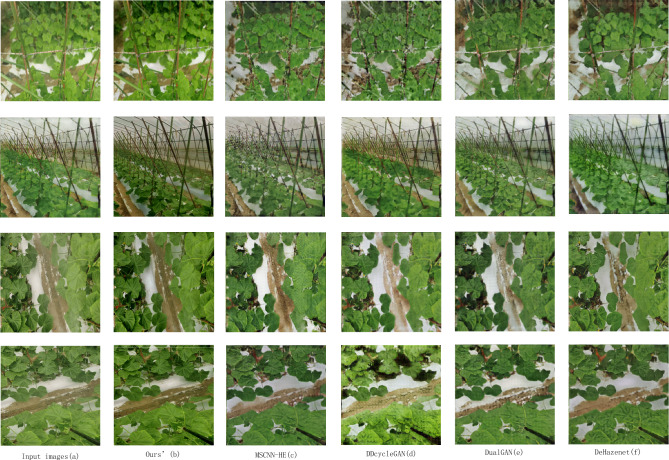
Comparison of the proposed algorithm with different algorithms in mist.

The comparison results of mist fog contrast are shown in Fig. 6.

**Figure 7:**
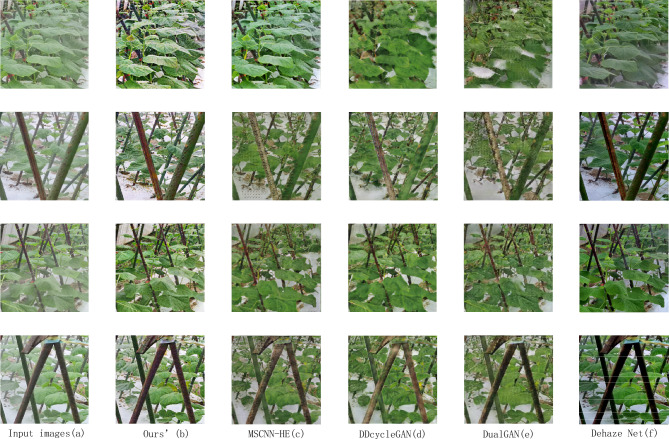
Comparison of the proposed algorithm with different algorithms in dense haze.

The comparison of thick fog contrast results is shown in Fig. 7.

Figure 7a shows the hazy image of the input network; Fig. 7b shows the proposed algorithm. Figure 7d and e shows the unpaired dehazing algorithms DDcycleGAN and DualGAN. Figure 7c and f shows the paired image algorithms MSCNN-HE and DehazeNet. From the training method of the data, we can see that the result of the paired image algorithm is better than that of the unpaired algorithm. However, the color of the results of the unpaired algorithm does not have a large deviation. In the dehazing results of DDcycleGAN and DualGAN, DDcycleGAN retains the contour information of the image well, but the texture information loss of the leaves is serious. The detailed texture generation of the leaves of DualGAN is better than that of DDcycleGAN, but there is a certain lack of leaf boundary contours. DehazeNet and MCSCNN-HE have served as excellent paired algorithms in recent years, and their good dehazing performance has been widely praised. However, in our study, the results of DehazeNet have color deviations, such as deepening the color of the iron in the figure and the deviation of the background color. MSCNN-HE avoids the color deviation of DehazeNet and has a good leaf outline. Moreover, some leaf lines are relatively clear, but there is still a certain gap between it and our proposed algorithm. The haze-free images generated in this study have no obvious color deviation, and the leaves' texture is clearly visible, providing accurate data for subsequent agricultural image processing.

Table 2 presents the EGF and FID metrics for the averaged images generated by various algorithms. From the data-level observation, we can see that the algorithm indicators proposed in this study are also prominent.

**Table 2:** Different algorithm result indicators.

	^Ours’^	^MSCNN-HE^	^DDCycleGAN^	^DualGAN^	^Dehaze Net^
^EGF^	^1,228.6^	^1,170.3^	^865.4^	^811.0^	^1,090.6^
^FID^	^46.78^	^53.99^	^81.44^	^85.27^	^61.63^

### The ablation experiment

**Figure 8:**
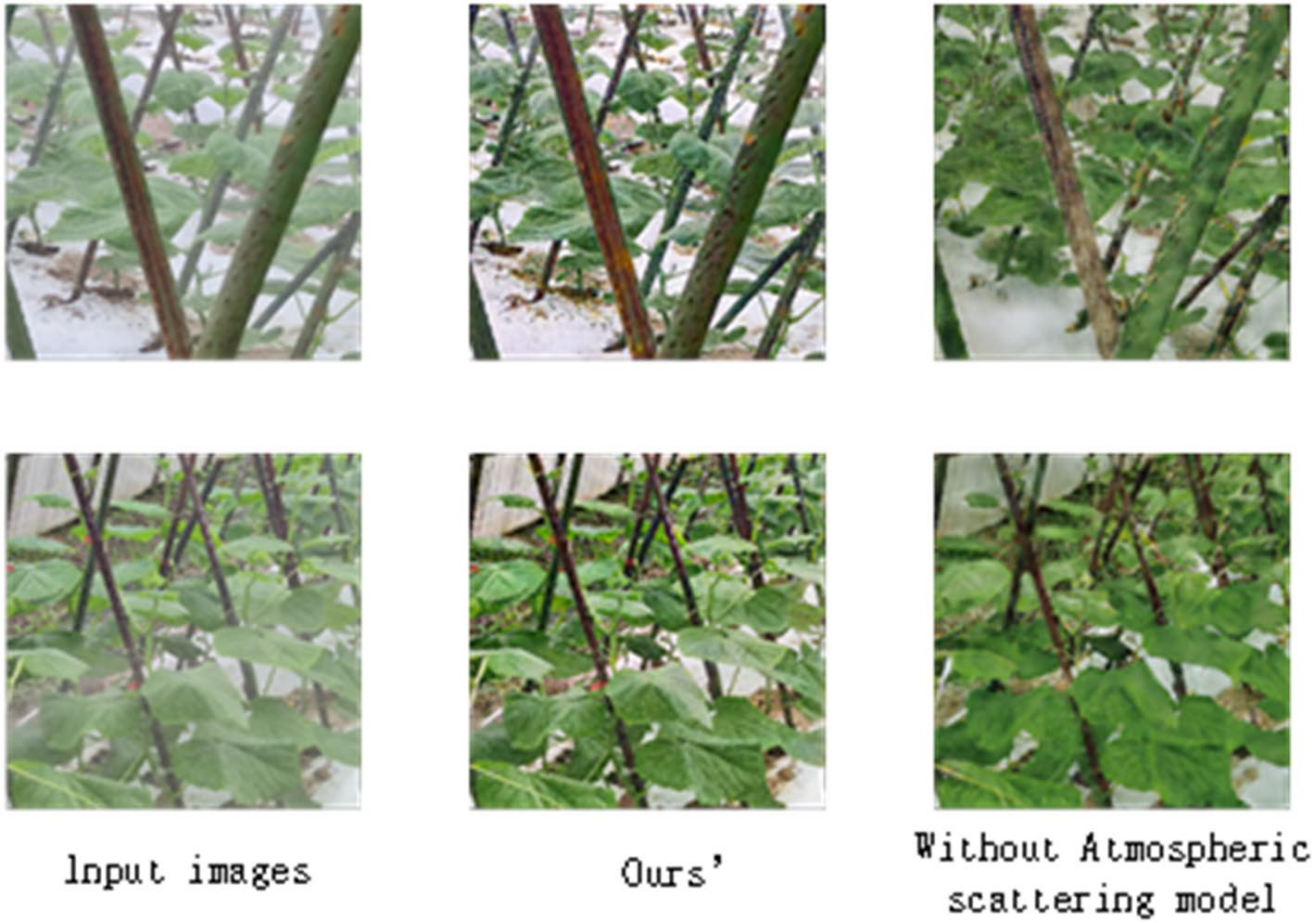
Comparison of between our’s method and Original GAN.

We compared the presence and absence of the atmospheric scattering model, and found that adding the atmospheric scattering model is more effective which shown in Fig. 8:

### Application

**Figure 9:**
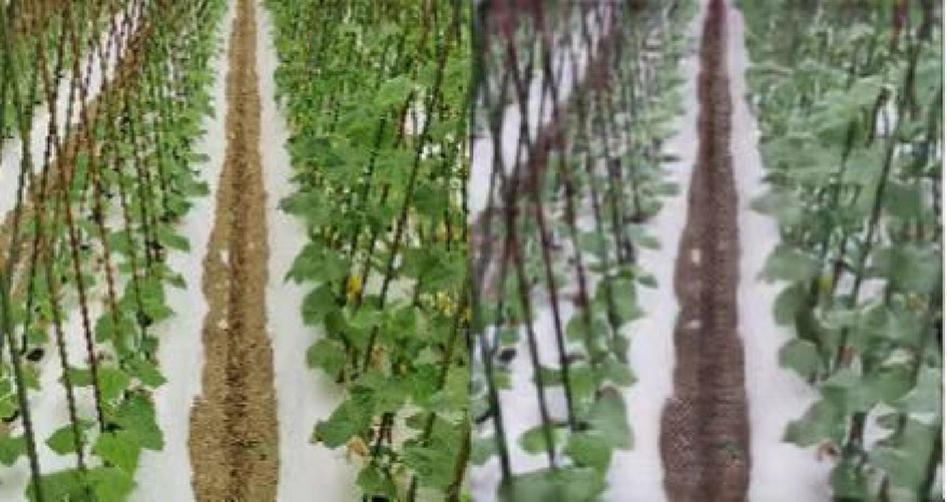
Comparison of between hazy images and haze-free images for application of blade shape determination.

**Figure 10:**
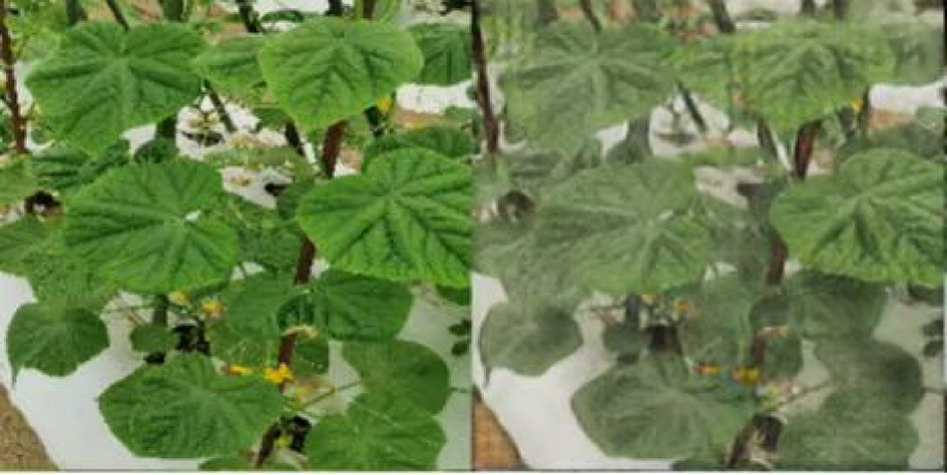
Comparison between hazy images and haze-free images for application of the cucumbers nutritional status determination.

The leaves of cucumbers are the main organs of photosynthesis, and there are significant differences in leaf size, shape, posture, color, and other aspects among different cucumbers. There are also differences between different varieties of the same cucumbers, such as palm shaped pentagon, heart shaped pentagon, nearly round, nearly triangular cucumber leaves. The leaf posture can also be divided into upright, flat, drooping, etc. Figures 9 and 10 compare the original images with and without the dehazing algorithm. As shown in Fig. 9, the original image on the right is blurry, the shape of the blade cannot be clearly seen. After dehazing, we can clearly determine that the blade shape is pentagonal heart shaped pentagon. Similarly, in Fig. 10, the original image is on the right side and the color of the leaves cannot be clearly seen. However, after dehazing, the nutritional status of the crop can be determined. Therefore, we can be confident that after dehazing, a complete and clear image of plant leaves can be obtained, which is helpful for further classification and identification of different cucumber and varieties.

## Conclusions

Aiming at the problem of obtaining more reliable phenotype information in hazy images for current precision agriculture, we proposed AgriGAN. First, we considered the cost and used unpaired image data for training. At the same time, we considered the method of combining traditional physics models with deep learning, adding atmospheric scattering models to the generator to reduce the difficulty of network dehazing and to improve network performance. Aiming at the problem that the efficiency of the discriminator in the generative adversarial network being lower than that of the generator, we proposed a discriminator model based on the detailed overall consensus identification to help the network reach the state of Nash equilibrium faster. Finally, the network achieved unpaired agricultural image dehazing through the addition of network adversarial loss + cycle consistent loss. The dehazing effect of the proposed algorithm was compared with that of existing dehazing algorithms. Our proposed algorithm is superior to the existing agricultural dehazing algorithms in terms of preserving image details and texture and reducing color deviation. Thus, it can effectively achieve high precision, reduce the cost of dehazing, and further the mission of image dehazing in the agricultural field.

### Additional information

The collection of plant materials complied with relevant institutional, national, and international guidelines and legislation.

## Data Availability

Our dataset and code will be publicly available at https://github.com/HZSUZJ/DLDF.

## References

[1] Shakoor N, Lee S, Mockler TC (2017). High throughput phenotyping to accelerate crop breeding and monitoring of diseases in the field. Curr. Opin. Plant Biol..

[2] Song P, Wang J, Guo X (2021). High-throughput phenotyping: Breaking through the bottleneck in future crop breeding. Crop J..

[3] Shi Y, Zhu Y, Wang X (2020). Progress and development on biological information of crop phenotype research applied to real-time variable-rate fertilization. Plant Methods.

[4] Bai G, Ge Y, Hussain W (2016). A multi-sensor system for high throughput field phenotyping in soybean and wheat breeding. Comput. Electron. Agric..

[5] Wang YH, Su WH (2022). Convolutional neural networks in computer vision for grain crop phenotyping: A review. Agronomy.

[6] Escamilla-García A, Soto-Zarazúa GM, Toledano-Ayala M (2020). Applications of artificial neural networks in greenhouse technology and overview for smart agriculture development. Appl. Sci..

[7] Chai J (2021). Deep learning in computer vision: A critical review of emerging techniques and application scenarios. Mach. Learn. Appl..

[8] Zhu H, Cheng Y, Peng X, Zhou JT, Kang Z, Lu S, Lim JH (2019). Single-image dehazing via compositional adversarial network. IEEE Trans. Cybern..

[9] Xu S, Wang J, Shou W (2021). Computer vision techniques in construction: A critical review. Arch. Comput. Methods Eng..

[10] Zhang X (2022). Research on remote sensing image de-haze based on GAN. J. Signal Process. Syst..

[11] Zheng Y, Su J, Zhang S (2022). Dehaze-AGGAN: Unpaired remote sensing image dehazing using enhanced attention-guide generative adversarial networks. IEEE Trans. Geosci. Remote Sens..

[12] Dong, Y., Liu, Y., Zhang, H., Chen, S., & Qiao, Y. (2020, April). FD-GAN: Generative adversarial networks with fusion-discriminator for single image dehazing. In Proceedings of the AAAI Conference on Artificial Intelligence (Vol. 34, No. 07, pp. 10729–10736)..

[13] Agarwal, I. (2022). Single Image Dehazing Using NN-Dehaze Filter. In International Conference on Innovative Computing and Communications (pp. 701–711). Springer, Singapore.

[14] Sun Z, Zhang Y, Bao F, Shao K, Liu X, Zhang C (2021). ICycleGAN: Single image dehazing based on iterative dehazing model and CycleGAN. Comput. Vis. Image Understanding.

[15] He K, Sun J, Tang X (2010). Single image haze removal using dark channel prior. IEEE Trans. Pattern Anal. Mach. Intell..

[16] Xian-li J, Wei Z, Lin-feng L (2020). Image defogging algorithm based on guided filtering and adaptive tolerance. J. Commun..

[17] Li, B., Peng, X., Wang, Z., Xu, J., & Feng, D. (2017). Aod-net: All-in-one dehazing network. In Proceedings of the IEEE International Conference on Computer Vision (pp. 4770–4778).

[18] Jin-sheng XIAO, Meng-yao SHEN, Jun-feng LEI (2020). Image conversion algorithm for haze scenes based on generative adversarial networks. Chin. J. Comput..

[19] Zhu, J. Y., Park, T., Isola, P., & Efros, A. A. (2017). Unpaired image-to-image translation using cycle-consistent adversarial networks. In Proceedings of the IEEE International Conference on Computer Vision (pp. 2223–2232).

[20] Zhao J, Zhang J, Li Z, Hwang JN, Gao Y, Fang Z, Huang B (2019). Dd-cyclegan: Unpaired image dehazing via double-discriminator cycle-consistent generative adversarial network. Eng. Appl. Artif. Intell..

[21] Wei Y, Zhang Y, Mei S (2017). Image dehazing method based on dark channel prior and interval interpolation wavelet transform. Trans. Chin. Soc. Agric. Eng..

[22] Zhang J, Wang X, Yang C (2018). Image dehazing based on dark channel prior and brightness enhancement for agricultural remote sensing images from consumer-grade cameras. Comput. Electr. Agric..

[23] Xiangpeng F, Jianping Z, Yan X (2021). Agricultural image dehazing method based on super-pixel dark channel and improved guided filtering. J. Agric. Mach..

[24] Ruowan G, Shuli M, Li Li, Aiping W (2019). Farmland image dehazing method based on wavelet precise integration and dark channel prior. J. Agric. Mach..

[25] Goodfellow Ian, J., Jean, P. A., Mehdi, M., Bing, X., David, W. F., Sherjil, O., & Courville Aaron, C. (2014). Generative adversarial nets. In Proceedings of the 27th International Conference on Neural Information Processing Systems (Vol. 2, pp. 2672–2680).

[26] Yi, Z., Zhang, H., Tan, P., & Gong, M. (2017). Dualgan: Unsupervised dual learning for image-to-image translation. In Proceedings of the IEEE International Conference on Computer Vision (pp. 2849–2857).

[27] Kim, T., Cha, M., Kim, H., Lee, J. K., & Kim, J. (2017). Learning to discover cross-domain relations with generative adversarial networks. In International Conference on Machine Learning (pp. 1857–1865). PMLR.

[28] Isola, P., Zhu, J. Y., Zhou, T., & Efros, A. A. (2017). Image-to-image translation with conditional adversarial networks. In Proceedings of the IEEE Conference on Computer Vision and Pattern Recognition (pp. 1125–1134).

[29] Ronneberger O, Fischer P, Brox T. U-net: Convolutional networks for biomedical image segmentation Medical Image Computing and Computer-Assisted Intervention–MICCAI 2015: 18th International Conference, Munich, Germany, October 5-9, 2015, Proceedings, Part III 18. Springer International Publishing, 2015: 234-241.

[30] Cai B, Xu X, Jia K, Qing C, Tao D (2016). Dehazenet: An end-to-end system for single image haze removal. IEEE Trans. image Process..

